# Responsible Learning About Risks Arising from Emerging Biotechnologies

**DOI:** 10.1007/s11948-021-00300-1

**Published:** 2021-03-29

**Authors:** Britte Bouchaut, Lotte Asveld

**Affiliations:** Department of Biotechnology, Section of Biotechnology and Society, Delft University of Technology, Van der Maasweg 9, 2629 HZ Delft, The Netherlands

**Keywords:** Risk management, Safe-by-design, Forward-looking responsibility, GMO regulation, Precautionary principle

## Abstract

Genetic engineering techniques (e.g., CRISPR-Cas) have led to an increase in biotechnological developments, possibly leading to uncertain risks. The European Union aims to anticipate these by embedding the Precautionary Principle in its regulation for risk management. This principle revolves around taking preventive action in the face of uncertainty and provides guidelines to take precautionary measures when dealing with important values such as health or environmental safety. However, when dealing with ‘new’ technologies, it can be hard for risk managers to estimate the societal or environmental consequences of a biotechnology that might arise once introduced or embedded in society due to that these sometimes do not comply with the established norms within risk assessment. When there is insufficient knowledge, stakeholders active in early developmental stages (e.g., researchers) could provide necessary knowledge by conducting research specifically devoted to what these unknown risks could entail. In theory, the Safe-by-Design (SbD) approach could enable such a controlled learning environment to gradually identify what these uncertain risks are, to which we refer as *responsible learning*. In this paper, we argue that three conditions need to be present to enable such an environment: (1) regulatory flexibility, (2) co-responsibility between researchers and regulators, and (3) openness towards all stakeholders. If one of these conditions would not be present, the SbD approach cannot be implemented to its fullest potential, thereby limiting an environment for responsible learning and possibly leaving current policy behind to anticipate uncertain risks.

## Introduction

In 2012, the scientific community was astounded when researchers discovered a new advanced gene-editing technique: CRISPR/Cas9. Due to its ability to edit almost any organism’s DNA material, it opened up many new possibilities for research and development (R&D) in a broad range of applications. For the fields of synthetic biology and biotechnology, this advanced technique means new possibilities for innovations using living organisms. But, it might also lead to more uncertain risks, i.e. ‘known unknowns’—although knowledge might still be limited, it does contain indications that a new type of event could occur in the future with possibly severe consequences (Flage & Aven, 2015). For biotechnology, these could range from technical issues (e.g., off-target mutations) (Gorter de Vries et al., 2019) to societal or environmental concerns (e.g., climate change or bioweapons) (Asveld et al., 2019; Ben Ouagrham-Gormley & Fye-Marnien, 2019). Although most debates concerning uncertain risks revolve around applications of non-contained (i.e. agricultural or ‘green’) biotechnology, this has also put emphasis on managing uncertain risks for contained (i.e. industrial or ‘white’) biotechnology, even though the associated uncertainties of these strands of biotechnology may differ, for example, their influence on natural ecosystems.

From a European risk governance perspective, currently, uncertain risks are covered in GMO-legislation by the embeddedness of the Precautionary Principle (PP). This ‘better safe than sorry’ principle provides guidelines for risk managers to take precautionary measures that are justified when dealing with important values such as threats to (societal) health or the environment (Sandin et al., 2002). An example of the embedded precautionary culture is the ruling of the European Court of Justice (ECJ) in the summer of 2018, that organisms treated with CRISPR-Cas technology should be classified as Genetically Modified Organisms (GMOs) (Purnhagen et al., 2018) in accordance with existing legislation on GMOs in the European Union (EU). Although CRISPR-Cas applications are still under development and therefore its associated risks are not completely known yet, the main concern arising from this ruling is the focus being too much on quantifiable risks instead of discovering what these uncertain risks might entail (Callaway, 2018). Considering the fast pace of developments and the associated uncertain risks within the field of biotechnology, a too strong focus on quantifiable risks might lead to a lack of incentive for researchers to discover and learn what these risks encompass, and possibly to a knowledge gap in risk governance to anticipate these unknown risks.

Given the normative approach and the embeddedness of the PP in GMO-legislation, room to learn what uncertain risks might entail is limited as uncertain risks do not meet the set norms. Effective risk governance should contain a flexible assessment procedure that takes into account that risks can be complex, uncertain and/or ambiguous (Hansson, 2016; van Asselt & Renn, 2011; van Asselt & Vos, 2008). But, to discover the complexity of uncertain risks responsibly, a controlled learning environment would be needed in which risks, step-by-step, can be identified. This gradual, controlled learning of what uncertain risks encompass is what we refer to as *responsible learning*. An approach that has been gaining attention over the past years and could enable such controlled learning is Safe-by-Design (SbD). As this approach aims to address safety issues already during early-stage development by stimulating engagement of a broad range of stakeholder iteratively (e.g., by feedback-loops), a variety of issues could already be brought up in these early stages of development and anticipated on in design choices made by researchers (Robaey, 2018; van de Poel & Robaey, 2017). For example, researchers, engineers, ecologists, biologists and policymakers might identify different (possibly long-term) issues that might come up during, and after a biotechnology has been developed. By already addressing and acting on these possible issues in the initial experimental design, these could be tackled early on. This would make it possible coming to a collective experimental design with safety in mind (Khan & Amyotte, 2003; van de Poel & Robaey, 2017). However, learning about uncertain risks would call for some regulatory flexibility since the established norms for known risks cannot be met during the set-up of an experiment as there is insufficient information available. Theoretically, the iterative character of SbD for making design choices can offer such flexibility to discover what these unforeseen risks possibly entail, in a responsible way. Also, this new information can help risk managers to amend the set norms of what is considered an acceptable risk to the state-of-the-art in biotechnology.

This paper addresses the following question: What conditions would be needed to enable an environment for responsible learning about new and uncertain risks of emerging biotechnologies? For the sake of clarity, although biotechnology can be classified in different ‘colours’ (i.e., red—biopharmaceuticals, white—industrial, green—agricultural), our study focuses on industrial biotechnology. In addition, although uncertain risks could emerge throughout a biotechnology’s development cycle (i.e. design-build-test-upscale-market phase), we focus on the early design stages up to building and testing as, ideally, we would want newly emerging risks to be anticipated on in the initial design choices of a biotechnology. Our study is structured as follows. First, we provide an overview of the current risk management regime in the Netherlands and the embeddedness of the PP, and the notion of forward-looking responsibility assigned to different groups of stakeholders, building upon van de Poel and Nihlen-Fahlquist (2012) (Sect. 3). Secondly, we analyse which conditions would be needed to create an environment suitable for responsible learning about uncertain risks. We identified the following three conditions: (1) regulatory flexibility, (2) co-responsibility between researchers and risk managers, and (3) openness towards all stakeholders (Sect. 4). These conditions are elaborated by using an illustrative discussion tool, i.e. a 3D cube (Sect. 4.2). Thirdly, we argue to what extent the SbD approach (Sect. 5) could provide guidelines for responsible learning and what would be required to do so considering the embeddedness of the PP in GMO legislation.

## Methods

This study comprises an empirically informed conceptual analysis of responsibility, uncertain risks and what would be needed to establish an environment for responsible learning. Literature studied for the conceptual analysis focused on the notion of forward-looking responsibility, the PP and its embeddedness in managing uncertain risks. For clarifying the context in which this study takes place, interviews were conducted with relevant stakeholders to gain insight in the current GMO permit application process from both a regulatory and practical perspective, how this relates to the interviewees’ perceived and assigned notion(s) of responsibility, and how we could or should take appropriate measures to anticipate uncertainties that might come along during the development of a biotechnology. From these interviews, three conditions were established that would be necessary to create an environment suitable for responsible learning. Based on literature, we discuss to what extent these conditions could be met by applying SbD, and whether this could be implemented considering the embeddedness of the PP in the risk management regime.

Interviews (N_tot_ = 9) were conducted between May and October 2019 and generally focused on two types of stakeholders. The first type is active as ‘applicants’—stakeholders who conduct experiments and are involved in risk assessment and permit application processes. These interviewees (N = 5) are employed as a principal investigator (PI), Postdoc researcher (PD), PhD researcher (PhD), technician/ designated responsible employee^1^ (DRE), and a Biosafety Officer (BSO^2^). The second type is active in the risk management stage (e.g., regulatory settings), consisting of interviewees (N = 3) employed by the Dutch GMO Office (BGGO^3^), and the Dutch Human Environment and Transport Inspectorate (ILT^4^). Also, one interview was conducted with an independent consultant, who could elaborate on the communication between applicants and risk managers.

The interviews followed a semi-structured approach that left enough room to go into detail when the researchers felt this was necessary for clarification or context. The interviewees were selected based on their experience in the domain of biotechnology and field of profession, and all hold senior positions, except for the PhD and Postdoc researcher. At the start of each interview, a form of consent was signed to approve recording of the interview. After the interview, a transcript was sent to the interviewee for any remarks or corrections. Upon receiving the interviewee’s approval, the transcript was coded and analysed. In terms of the conceptual analysis, since this takes place within a specific context (i.e., contained use of industrially applied biotechnologies), the empirical input from the interviews helped to clarify relevant concepts for this study. Lastly, 3 out of 9 interviews were conducted in English, the others were conducted in Dutch. Therefore, some quotations in-text have been translated into English^5^. In addition, although all information provided by the interviewees is based on Dutch GMO legislation, it is still relevant to other countries or EU Member States. Albeit there are some legislative differences within the EU, all States have to adhere to the uniform EU directives. Also, issues associated with discovering uncertain risks responsibly are everywhere at stake, also outside the EU.

## European Risk Governance

The regulation of biotechnologies within the EU has been active since the 1990s. The main principle that underlies EU legislation is the PP which originated in German domestic law during the 1970s and 1980s and has been incorporated in many international environmental treaties and agreements since then (Berg et al., 1974; Jelsma, 1995; Marchant & Mossman, 2004). In 1992, the EU committed to conform their environmental policy with the PP in the Maastricht amendments (Article 174.2^6^). Later, EU policy was followed by many individual European nations (e.g., Germany, France and the Netherlands), leading to an integrated ‘precautionary process’ within risk assessment and management (Stirling, 2007). In 2003, European GMO legislation was complemented with the Cartagena Protocol on Biosafety. Particularly the safe distribution of GMOs between countries is emphasized in this protocol, but it also addresses possible adverse effects on the conservation and sustainable use of biological diversity, and risks to human health (Kinderlerer, 2004).

In terms of the PP, there has been disagreement about its measures for biosafety and in terms of its effectiveness. For more than a decade, opponents have argued that the way the PP is embedded in the regulatory regime is confusing (Manson, 2002), too complex (Sandin et al., 2002), primarily designed to stop the use of modern biotechnology—hindering Europe to realise the benefits that biotechnology could bring (Kinderlerer, 2004), or implemented differently than originally intended (Tagliabue, 2015, 2016) and an update would be required (Hansson, 2016). More recently, proponents have argued that the PP can confine risks, although it can restrict opportunities as well (Anyshchenko, 2019), or that it is a way to use information about known risks in the best possible way and can help to create public acceptance of biotechnologies (European Union, 2017; Taleb et al., 2014).

Although the PP inherently can provide researchers and risk managers with guidelines to avoid taking unnecessary risks, the way this principle is operationalised now appears to withhold them from exploring what any ‘new’ uncertain risks might entail. Considering the fast developments in biotechnology, there needs to be room to explore what uncertain risks these might bring, to learn what these entail and how to anticipate these responsibly. In the Netherlands, although certain procedures allow researchers to explore these uncertainties to some extent, the way the PP in currently embedded does not stimulate conducting such research.

### National Risk Governance

In the Netherlands, risk governance is influenced by many different groups of stakeholders, e.g., the European Commission, Dutch Parliament, citizens, researchers, etc. However, from an executive perspective, risk governance processes generally involve three groups of stakeholders: risk managers, risk assessors, and applicants. *Risk managers*, e.g., policymakers or regulators, focus on the management of risks which comprises more normative questions e.g., how ‘big’ these uncertain risks are? or how acceptable these would be, and to whom? (Asveld, 2007; Asveld et al., 2019). In other words, risk managers set the norms for risk assessments; a measure for the normative conception of risks. *Risk assessors* determine whether a submitted risk assessment meets the set norms, e.g., are the risks involved in an experiment acceptably safe? (Kermisch, 2012). Although this group of stakeholders is active within Governmental organizations (e.g., the Dutch GMO Office or BGGO), they are not considered regulators. *Applicants*, e.g., researchers and BSOs, must conduct a risk assessment and submit these to the designated governmental agency before starting their experiments. When dealing with uncertain risks, quantitative data for risk assessment can remain incomplete or limited due to lack of experience or knowledge (Collingridge, 1982; Genus & Stirling, 2018). As risk managers and risk assessors generally do not conduct experimental research themselves, it can be difficult for them to determine *when* and *how* to act on uncertain risks appropriately and responsibly. Therefore, stakeholders who do conduct experiments, i.e., researchers, could provide necessary knowledge by devoting research specifically to what these uncertain risks could entail (Linkov et al., 2018). For the sake of clarity, although we acknowledge the differences in executive duties pertaining to risk managers and risk assessors, within this paper we will refer to both groups of stakeholders as *Risk Managers*. Those who conduct risk assessments (e.g., researchers and BSOs) are referred to as *applicants*.

#### Procedure

**Fig. 1:**
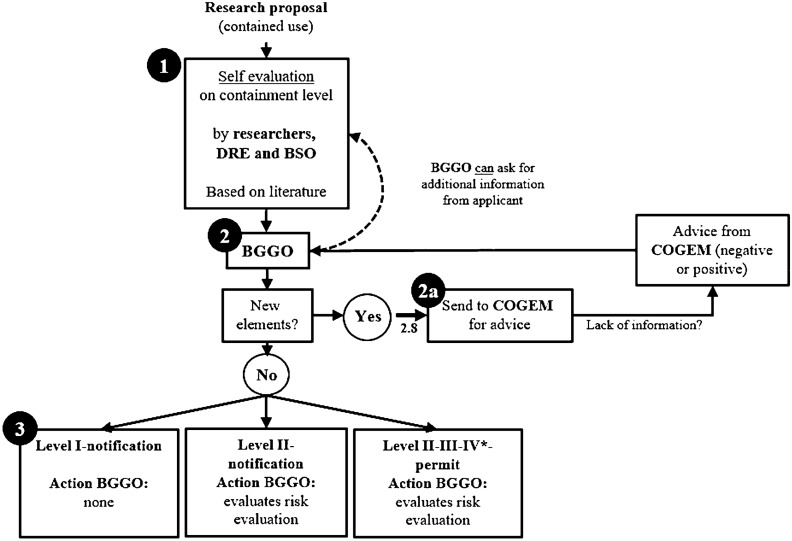
Schematic representation of the Risk Governance system for GMOs (contained use) in the Netherlands. Illustration produced in collaboration with Rathenau Instituut, The Hague, the Netherlands

Based on all interviews, we have derived a schematic representation of the risk assessment and permit application procedure for contained use of GMOs in the Netherlands—see Fig. 1. At the start of this procedure, researchers are requested to do a risk assessment in which already set norms by risk managers are embedded, for example, societal or environmental implications of components. The risk assessment is conducted by the involved researchers and BSO and is often based on literature or previous permit applications (Fig. 1—level 1). When no new components or elements are introduced, the risk assessment procedure automatically assigns an appropriate Biosafety Level (BSL) and a corresponding Microbiological Laboratory (ML) class in which researchers can conduct their experiment (Fig. 1—level 3). In this case, researchers must only make notification of their experiment(s) to BGGO (applicable for BSL I-II). When there are new elements introduced or the assigned BSL is above level II-notification, researchers must request a permit from BGGO. However, due to the ‘newness’ of such elements or technologies, there might be inadequate knowledge about the technology’s possible risks at that moment. When such a situation occurs, BGGO could prohibit the experiment, examine whether it is possible to approve an experiment but with a higher BSL (e.g. level IV^7^) and additional conditions, or could ask the applicants to adjust their experimental set-up so it would fit the current BSL-norms. In response, applicants can apply for the so-called ‘2.8 procedure^8^’ (Fig. 1—level 2a) when they believe that, for example, a lower containment level would also be sufficient or when they do not have access to a laboratory with the assigned ML-class. When such procedure becomes active, applicants have to provide more information which shows that a lower level would also be acceptable. Based on this, risk managers from BGGO (Fig. 1—level 2a) can ask the Commission on Genetic Modification (COGEM) for advice. Based on this, BGGO could approve the 2.8 application with a lower designated BSL, or reject it and remain to the initially assigned BSL. In response, researchers can follow this decision, or adjust their experiment and go through the process again by filing a new application.

#### Procedure in Practice

On March 1st 2015,^9^ a change was implemented regarding the GMO procedure. Where prior to this date the risk assessment procedure was conducted by BGGO, from this date on, research institutions have the responsibility to carry out this assessment themselves and have to determine whether additional regulations apply (only for level I and level II-notification). This renewal was based on vast experience with historical permit requests and aimed to lighten the administrative burden for institutions. Interviewees (PI; PD; PhD: BSO) acknowledge that it has accelerated research as they do not have to await formal approval, but it has also made the risk assessment a slightly routinized procedure. Interviewed risk managers (BGGO1; BGGO2), however, acknowledge that the workload for researchers has increased as a result of this renewal. Furthermore, interviewee PhD also mentions that when ‘new’ elements are introduced and additional information is requested by BGGO, the decision-making process of whether this would be acceptably is also dependent on the researchers’ input, which implies them to also have responsibility. “*We have all kind of micro-organisms and viruses in here. So they [BGOO] don’t know what biosafety level [to assign]. I have level 1 micro-organisms, but also level 3. […] so basically, all I had to say was ‘Okay, biosafety 3 level micro-organisms are really low in terms of abundance’. I just had to present it and prove that nothing dangerous is happening here” (PhD).* Taking into account that the executive risk assessment for level I and II-notification also lies with researchers, trust and openness become important for responsible risk management. Although there is currently little reason to question researchers’ integrity, we should still be alert that this does not lead to a false sense of safety. *“Yes, for [level] 2 and 3, the risk assessment is carried out by the institution and BGGO checks it. Not for [level] 1, this level is let go* [sic] *as there is also lower risk and we do not assume that people [researchers] who work on level 1 should actually work at level 3. We don’t have that feeling” (ILT).*

Ideally, researchers should take safety and possible emerging risks already into account during the early developmental stage of a biotechnological product or process and (re)consider their design choices accordingly, e.g., while composing a research proposal. However, the way the PP is currently being operationalized in risk assessment procedures appears to withhold relevant actors from exploring any new, uncertain risks. If such risks are arising, researchers are asked to adjust their experimental set-up (or safety level) so it fits the current BSL norms. In that sense, we can say that the established risk management regime is a regime of compliance. Researchers are assigned a form of forward-looking responsibility to prevent risks from occurring, but no such responsibility is assigned to them for knowing, assessing and communicating uncertain risks.

### Forward-Looking Responsibility

Forward-looking responsibility entails measures that aim for that something *does* happen—taking appropriate preventive measures (van de Poel, 2011). Therefore, in terms of preventing risks from occurring, forward-looking responsibility plays a crucial role in dealing with uncertain risks and exploring what these specifically entail. Building upon forward-looking responsibility for risks as described by van de Poel and Nihlen-Fahlquist (2012), we subdivide this notion into four main categories. These are (1) Responsibility for risk reduction, (2) Responsibility for risk assessment, i.e. establishing risks and their magnitude, (3) Responsibility for risk management, including decisions about what risks are acceptable and the devising of regulations, procedures and the like to ensure that risks remain within the limits of what is acceptable, and (4) Responsibility for communication about risks (van de Poel & Nihlen-Fahlquist, 2012). However, considering the rapid developments in the field of biotechnology, we believe that the notion of forward-looking responsibility should not only comprise ‘known’ risks, but also *uncertain* risks. In the following section, we first provide an overview of which subcategory of forward-looking responsibility can currently be assigned to risks managers and applicants and argue that the forward-looking responsibility for knowing, assessing and communicating about uncertain risks is not specifically assigned to either of them.

#### Forward-Looking Responsibility

*Risk managers* are forward-looking responsible for establishing the norms of what would be acceptably safe, and what would not. Therefore, risk management involves questions of values, e.g., what is safe ‘enough?’ and is based on a trade-off of what would be considered acceptably safe and what not e.g., assigning an appropriate BSL. In the Netherlands, current legislative settings for biotechnology can be described as a precautionary culture where the Dutch government is held accountable for inducing risks towards society or the environment, even unknown risks (Helsloot et al., 2010), thereby assuming that research facilities or industry have complied with regulation. In addition, as risk managers are also involved in assessing and anticipating uncertain risks, they can also be ascribed a form of forward-looking responsibility which refers to making sure the ‘right’ precautionary measures are taken to anticipate any uncertain risks.

Within current regulation, *applicants* (i.e., researchers, BSOs or designated responsible employees) are also assigned forward-looking responsibility. As already touched upon in the previous section, applicants have the responsibility to do a risk assessment before the start of their experiment(s) meaning that they must make design choices based on the established norms by risk managers. However, in terms of uncertain risks—also possible unforeseen issues that may arise while already conducting experiments, the assigned responsibilities are unclear. Table 1 summarises the assigned forms of forward-looking responsibility of risk managers and applicants for known and uncertain risks and illustrates that currently, there is no assigned form of forward-looking responsibility for knowing, assessing and communicating uncertain risks.

**Table 1:** Subcategories of forward-looking responsibilities assigned to risk managers and applicants (known and uncertain risks), built upon van de Poel and Nihlen-Fahlquist (2012)

Subcategory of forward-looking responsibility	Known risk	Uncertain risk
Responsibility for setting standards for acceptable risks	Risk managers (e.g., BSL levels)	Risk managers (precautionary principle)
Responsibility for knowing and assessing risks	Risk managers & applicants	*Not assigned*
Responsibility for reducing risks	Applicants	Applicants
Responsibility for communication about risks	Risk managers & applicants	*Not assigned*

## From Compliance to Responsible Learning

Within this study, we refer to *responsible learning* as stimulating researchers to proactively (re)consider their experimental design choices for the sake of safety, while also being able to explore what any uncertain risks might entail. As discussed in the previous section, the current risk management regime seems to be one of compliance in which no forward-looking responsibility is assigned to researchers for knowing, assessing and communicating uncertain risks. But, considering the fast pace of biotechnological development, (more) uncertain risks can be expected to arise. Therefore, ideally, researchers should proactively identify and anticipate uncertain risks and (re)adapt their experimental design by taking appropriate measures. In this section, we argue that three conditions should be met to create an environment for responsible learning, derived from the not assigned forms of forward-looking responsibility (see Table 1): regulatory flexibility, co-responsibility and openness. An overview of the derived conditions needed for responsible learning is provided in Table 2.

**Table 2:** Overview of the not-assigned forms of forward-looking responsibility to researchers and risk managers, and the derived conditions needed for responsible learning

Subcategory of forward-looking responsibility	Known risk	Uncertain risk	Derived conditions for responsible learning
Responsibility for setting standards for acceptable risks	Risk managers (e.g., BSL levels)	Risk managers (precautionary principle)	*Regulatory flexibility*
Responsibility for knowing and assessing risks	Risk managers & applicants	*Risk managers & applicants*	*Co-responsibility*
Responsibility for reducing risks	Applicants	applicants	*Co-responsibility*
Responsibility for communication about risks	Risk managers & applicants	*Applicants*	*Openness*

### Conditions for Responsible Learning

Although experiments should always be designed and executed with caution, the degree of uncertain risks can only become known by experimenting and learning. Therefore, we introduce the first needed condition for responsible learning: *regulatory flexibility*. Recalling current GMO regulation and biosafety rules for confined use of GMOs within the Netherlands as described in Sect. 3, experimental designs need to be determined before conducting a risk assessment. When there are too many uncertainties in terms of risks, researchers are asked to provide more information concerning the elements of their experimental design. If there would be no or little literature available, this can create complications in terms of approving the experiment and the possibility of exploring and learning about possible uncertain risks. Therefore, we should transition to a more balanced form of risk management in which there is more room (and more stimuli) to discover what uncertain risks entail, in a responsible way. However, in addition to regulatory flexibility, two other conditions would be crucial for anticipatory, inclusive and responsive learning about new and uncertain risks: co-responsibility and openness.

As touched upon earlier, trust and openness appear to be important matters regarding safety. Interviews conducted with researchers and academic staff (PI, PD, PhD, BSO, DRE) indicate that they do perceive a strong responsibility in this respect. They mention that, based on their experience and awareness of the state-of-the-art in biotechnology, they should be capable of estimating whether an experiment might come with any unforeseen risk and whether these would be acceptable or not. However, they argue that the norms established within the current risk assessment are becoming outdated due to the fast developments within this field (Bouchaut & Asveld, 2020), and a regulatory update would be necessary. They also point out that they need room to explore uncertain risks to take on more forward-looking responsibility but that this is sometimes stifled by current regulation and regulatory practices. So, if researchers would also be assigned a form of forward-looking responsibility—creating *co-responsibility*—both parties can see to it that the ‘right’ measures are taken to prevent any harm done while it would also contribute to researchers carefully (re)consider their experimental design choices based on them also being accountable (though not in a legal sense) (van de Poel & Robaey, 2017).

However, some of the interviewed risk managers indicate that they believe that researchers might have different motives to conduct their research and do not always prioritize safety to the same extent, thereby complicating stakeholders having co-responsibility. “*I think there are different kinds of researchers, some are operating on a more fundamental level. […] If you continue such research and try to answer such related questions, it’s not so much about ‘how do you design something inherently safe?’. Well no, one wants to know an answer to that question and if that can be done quickly *via* a [biosafety]level 3 way, then they do it *via* a [biosafety]level 3 way. Another type of researcher, if they already have an application in mind, something like ‘I want to make a vaccine’, then they already know ‘my vaccine must be safe’, otherwise, it will never enter the market” (BGGO1)*. In that sense, both parties having co-responsibility calls for *openness*—the third condition needed for responsible learning. Researchers should be open and responsive towards risk managers or any other associated stakeholders about any unclear or ambiguous experimental results (Sonck et al., 2020). Also, both parties trusting each other would be of great importance here. By early addressing unforeseen issues and opening up a dialogue, these issues can be anticipated on and appropriate measures can be taken of which risk governance can also benefit. However, practically, we do not envision these interactions to happen for every step taken within the product development process. Instead, researchers should structurally reflect on the decisions to be made, thereby applying perspectives of other stakeholders by means of, for example, having occasional awareness exercises. A culture should be established where this is ‘common practice’ as has been happening in, for example, healthcare and the aviation industry (Singh, 2009), or more recently, in the field nanotechnology (Rerimassie et al., 2018).

In summary, to find a balance between taking appropriate precautionary measures and ‘proceeding with caution’, some regulatory flexibility would be needed for researchers to be able to discover what uncertain risks might entail. Also, researchers should actively (re)consider their design choices for the sake of safety which can be stimulated by assigning them co-responsibility (van de Poel & Robaey, 2017). However, as Bouchaut and Asveld (2020) point out, perceptions of risks and safety tend to differ between stakeholders which can be based on their worldview, experience, or professional position (De Witt et al., 2017). As researchers find themselves in a different position than risk managers, their perspective on what would be an acceptable risk might differ. Besides, researchers holding different positions (i.e., PIs, Postdocs or PhD researchers) might also have a different perception of what would be acceptably safe and whatnot, due to their professional experience. Therefore, researchers must be open towards other stakeholders about their experimental findings or any unforeseen issues they might foresee or might arise during the experiments. But, the conditions we have identified to enable responsible learning can occur in different degrees. For example, how open should researchers be about issues they might expect but haven’t encountered yet as this could cause unnecessary turmoil? In the section below, some ‘extreme’ scenarios are elaborated, illustrating that all conditions should be present to some extent to enable an environment for responsible learning.

### Scenarios for Responsible Learning

**Fig. 2:**
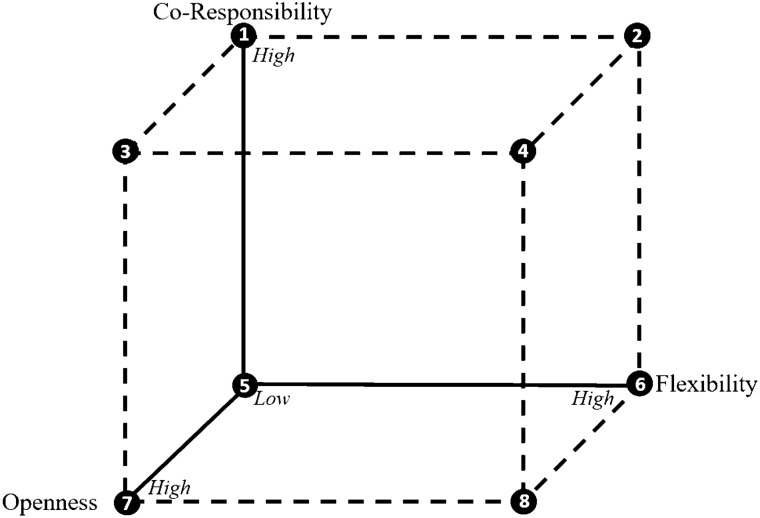
Graphical representation of the ‘low’ or ‘high’ degrees of openness, (regulatory) flexibility and co-responsibility (X, Y, Z-axis) needed to enable an environment suitable for Responsible Learning

We can establish different scenarios where the three identified conditions for responsible learning would have a ‘low’ or ‘high’ degree. In this paper, we use a 3D cube (Fig. 2) as a discussion tool where each axis represents one of the three conditions, clearly illustrating a specific scenario that might occur when differing degrees of these conditions would be present. As we would like researchers to proactively (re)consider their design choices in early experimental settings, the needed conditions for responsible learning are argued from an applicant’s perspective. Therefore, the condition of *co-responsibility* between risk managers and applicants is illustrated as a shared degree of forward-looking responsibility to assess and reduce risks, *regulatory flexibility* in terms of the normative assessment of risks, and *openness* as the extent researchers should be open about their experimental findings, and to what extent they would be aware of potential implications of their experiment by e.g. incorporating various stakeholders during the experimental design phase and including their perspectives in design choices.

As mentioned earlier, having all conditions present in some degree would be prerequisite for an environment for responsible learning. Would one of the conditions not be present, or only in low degrees (e.g., vertexes 2, 3 or 8), this would limit responsible learning. Vertex 2 illustrates both co-responsibility and regulatory flexibility to be present, but no, or only little openness and responsiveness of researchers concerning their experiments’ potential implications. As this could result in researchers applying for potentially ‘high-risk’ experiments, the high degree of regulatory flexibility may also lead to them being approved. Although this would already go against our understanding of responsible learning or responsible behaviour in general, this could also result in an extra burden for risk managers. Also, as there would be co-responsibility between researchers and risk managers, the latter would be accounted blameworthy might this have detrimental effects on society or the environment. Considering these could be ‘high-risk’ experiments, there is a chance this will be the case. Vertex 3 illustrates high degrees of openness and co-responsibility, but no, or only a low degree of regulatory flexibility. If there would be only very little room within regulation to learn what uncertain risks might entail, and which could also take up considerably more time in terms of getting their experiments approved (i.e. 2.8 procedure), it would sound superfluous for researchers to specifically devote experiments to this type of research, nor would assigning a form of responsibility to researchers seem reasonable. Vertex 8 illustrates both openness and regulatory flexibility being present, but no co-responsibility. In this case, researchers would not be held accountable which could incite them to be less open about potential uncertain risks, which would not contribute to researchers designing their experiments more responsibly.

Although all conditions should be present, not all should be met at their highest degree. For example, vertex 4 illustrates all three conditions present in their highest degree. Considering the condition of openness, not only would this seem not feasible (i.e., when can you be fully open of matters you possibly cannot know yet or may only slightly expect?), it might also hinder researchers from conducting research. When researchers would be ‘fully’ open about all possible consequences their research might have, would it still be worth the time and effort to research these? This scenario also applies to vertex 7, where only openness is present in the highest degree, compared to low degrees of regulatory flexibility and co-responsibility.

## Safe-by-Design for Responsible Learning

Recalling our definition of responsible learning and the defined conditions to enable such an environment, in theory, the SbD approach could provide guidelines for a controlled, iterative, step-by-step exploration of what uncertain risks could consist of, what consequences they might have, and how to anticipate these accordingly. This section first briefly explains the SbD approach and thereafter elaborates how this approach could provide guidelines for the earlier identified conditions needed for responsible learning. Secondly, we argue to what extent implementing SbD would be hindered by the embeddedness of the PP in the current risk management regime.

### Safe-by-Design

**Fig. 3:**
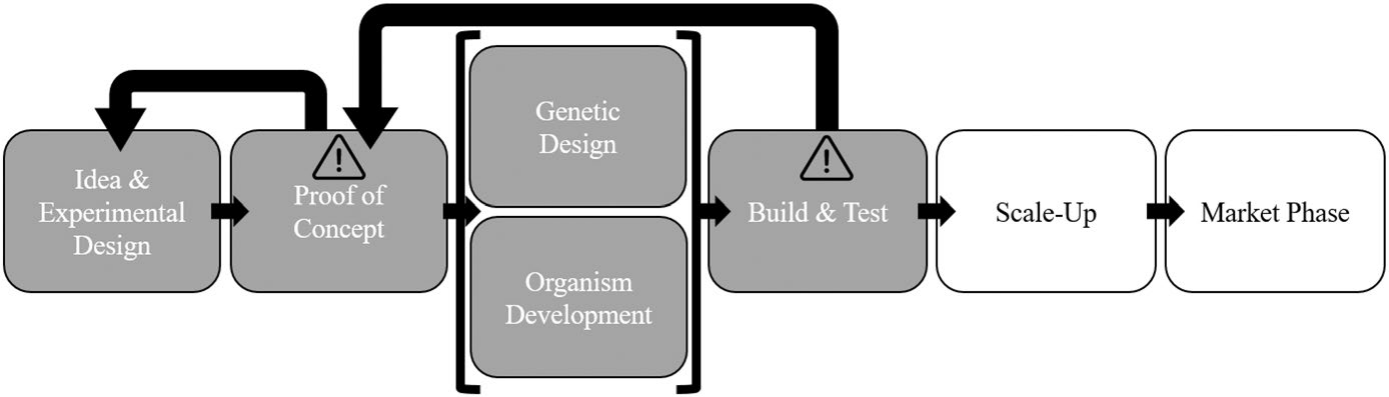
Schematic illustration of a (simplified) biotechnology’s development process, and the iterative character of Safe-by-Design. The parts from ‘scale-up’ onwards (in white) are left out of consideration as the focus within this study is on experimental design choices and nog upscaling or market implementation

SbD is an approach that comprises both engineered and procedural safety by “using materials and process conditions which are less hazardous” (Bollinger et al., 1996; Khan & Amyotte, 2003) and finds its origin in the domain of chemical engineering. More recently, this approach has gained attention in the fields of nanotechnology (Kelty, 2009; Kraegeloh et al., 2018; Schwarz-Plaschg et al., 2017), synthetic biology (Asin-Garcia et al., 2020) and biotechnology (Robaey et al., 2017; van der Berg et al., 2020). The SbD approach is associated with learning processes that aim for designing specifically for the notion of safety by iteratively integrating knowledge about the adverse effects of materials (van de Poel & Robaey, 2017). In particular the iterative character (i.e., feedback loops) of this design approach and the inclusion of a wide range of stakeholders could provide a way to gradually discover uncertain risks (Bouchaut & Asveld, 2020). By including different stakeholders throughout the experimental design process, different issues can be identified and active anticipation of possible risks is stimulated. This might broaden the scope of possible risk-related issues, and could impact experimental design choices. For example, may a possible risk be identified during the Build & Test phase, one could go steps back in the design process, and try to anticipate these beforehand by making adaptations in the design choices (Fig. 3). By implementing these so-called feedback loops, eventually, a collective ‘safe’ design could be achieved. Still, when dealing with emerging biotechnologies, it can become difficult to foresee future implications of the technology due to researchers’ lack of experience or the biotechnology could be used differently than devised (Collingridge, 1982).

### Guidelines for Responsible Learning

In terms of the earlier identified conditions needed for responsible learning, the SbD approach could provide guidelines to enable these—in particular the conditions of co-responsibility and openness. In terms of regulatory flexibility, SbD cannot enable such, but it could help to monitor such a more flexible risk management regime.

The conditions of co-responsibility and openness could be met to a certain extent by applying the SbD approach. The SbD approach stimulates active stakeholder participation, openness and responsiveness, creating a dialogue between parties where we can reach agreement on what would be considered acceptably safe and what measures would be appropriate to take. Might any unforeseen (and unacceptable) issues arise, researchers could anticipate these in their experimental design accordingly. This iterative character also provides flexibility in terms of design, however, no regulatory flexibility. Still, we believe that the SbD approach could be a suitable candidate for responsible learning, provided that regulation would allow so. However, one of the challenges would then also become how to monitor these types of research devoted to exploring uncertain risks in a proper way. Assigning researchers co-responsibility to assess and reduce uncertain risks might help tackle this challenge.

### Barriers for Safe-by-Design

Recalling the different ‘extreme’ scenarios described in Sect. 4.2, this illustrates that the conditions of regulatory flexibility, co-responsibility and openness should all be present, but to some degree. However, placing these findings in the perspective of the current embeddedness of the PP in GMO regulation, and the focus herein on quantifiable risks, the condition of regulatory flexibility cannot be met to the desired degree (Hansson, 2016; Stirling et al., 1999). Recalling Sect. 3.1.1, when dealing with ‘new’ elements, the 2.8 procedure can come into force. The advice of COGEM and the decision-making of BGGO is mainly based on literature—albeit partly provided by the applicants. Although depending on the ‘newness’ of these elements or processes, when there is no sufficient literature available, this leads to different scenarios; researchers are required to provide more information (which would not be possible in this case), or given the option to reconsider their experimental set-up and adjust these accordingly so it does meet the set standards and an appropriate BSL can be assigned. So, when the set-up of an experiment would already be prohibited due to the risks being ‘too’ uncertain, this would limit room for learning what these uncertain risks exactly are. In other words, when no research can be conducted to exploring what uncertain risks entail, no literature can be devoted to these matters, leading to a vicious circle where research devoted to exploring uncertain risks is obstructed. As a result, the SbD approach cannot be implemented to its fullest potential, specifically the iterative character of SbD to anticipate uncertain risks.

## Conclusion

This study explored what conditions would be needed to enable an environment for responsible learning about new and uncertain risks of emerging (white) biotechnologies. First of all, we described the risk management regime in the Netherlands and argued that this is currently a regime of compliance in which researchers are assigned forward-looking responsibility to prevent risks from occurring, but not for knowing, assessing and communicating uncertain risks. Therefore, there is a need to create room for exploring uncertain risks and thus to create conditions for anticipatory and responsible learning about these risks.

To enable an environment suitable for responsible learning, we identified three conditions that should be met: (1) regulatory flexibility, (2) co-responsibility between risk managers and applicants, and (3) openness.

Lastly, we analysed how the SbD approach could provide guidelines for responsible learning in a controlled, iterative and step-by-step fashion and for considering design choices accordingly. In terms of the established conditions, SbD can provide a framework for co-responsibility and openness by active stakeholder engagement and the iterative character of SbD. Might any unforeseen (and unacceptable) issues arise, researchers could anticipate these in their experimental design choices. This iterative character also provides flexibility in terms of design, however, no regulatory flexibility. Still, we believe that the SbD approach could be a suitable candidate for responsible learning, provided that regulation would allow so. However, one of the challenges would then also become how to monitor these types of research devoted to exploring uncertain risks in a proper way. Assigning researchers co-responsibility to assess and reduce uncertain risks might help tackle this challenge. Also, stimulating openness and responsiveness amongst researchers about their experimental findings—after and during their experiments—could also help both groups to gain more trust in each other.

We are not advocating that researchers should have the freedom to take unacceptable risks. But, we believe that responsible learning could be a way for researchers to design experiments more responsibly, while also stimulating research specifically devoted to exploring uncertain risks of which risk governance can benefit as well.

## Limitations

This study was carried out within the Netherlands and focuses on Dutch legislation regarding contained use of GMOs. However, as the issue of managing and anticipating newly emerging risks is at stake globally, we believe that our findings are not limited to the Dutch context only. In terms of EU policy, although we acknowledge that there are differences between the EU Member States, all have to adhere to the uniform EU directives. Also, the conditions we defined to enable an environment for responsible learning are not necessarily bound to EU legislation (on which Dutch legislation is based), and could therefore be applied to other contexts. However, as our analysis of the applicability of the SbD approach is based on the embeddedness of the PP in EU legislation, this cannot be generalized outside the EU.

## Data Availability

All data can be accessed via the Electronic Archiving System (EASY) provided by the Royal Netherlands Academy of Sciences (KNAW) and the Netherlands Organization for Scientific Research (NWO) via https://doi.org/10.17026/dans-x9u-g6u4.

## Footnotes

1 In Dutch: verantwoordelijk medewerker. This employee assists in conducting risk assessments and is seen as the ‘bridge’ between researchers and the Biosafety Officer.

2 Biosafety Officers (BSOs) have a coordinating, motivating and signalling responsibility for biological safety when working with GMOs. BSOs provide support to researchers and research staff by providing information and advice on experimental designs and licence applications from the BGGO.

3 In Dutch: Bureau Genetisch Gemodificeerde Organismen (BGGO).

4 In Dutch: Bureau Inspectie Leefomgeving en Transport (ILT).

5 All data (i.e. form of consent, interview protocol, transcripts) can be requested from the corresponding author via https://doi.org/10.17026/dans-x9u-g6u4..

6 https://eur-lex.europa.eu/LexUriServ/LexUriServ.do?uri=CELEX:12002E174:EN:HTML.

7 There are no level IV laboratories in the Netherlands.

8 https://www.ggo-vergunningverlening.nl/ingeperkt-gebruik/procedures/bijzondere-procedures.

9 https://www.infomil.nl/onderwerpen/integrale/omgevingsloket/versies/wijzigingen-eerdere/versie-2-11/versie-2-12/inhoudelijke/besluit-regeling-ggo/.
